# Thyroxine Therapy Treating Infertility and Pituitary Tumor: Primary Hypothyroidism Leading to Pituitary Hyperplasia

**DOI:** 10.7759/cureus.60135

**Published:** 2024-05-12

**Authors:** Sidra Aslam, Ahmed Imran Siddiqi, Waqas Shafiq, Wania Rafaey

**Affiliations:** 1 Endocrinology, Shaukat Khanum Memorial Cancer Hospital and Research Centre, Lahore, PAK; 2 Endocrinology and Diabetes, Shaukat Khanum Memorial Cancer Hospital and Research Centre, Lahore, PAK; 3 Internal Medicine, Liaquat National Hospital, Karachi, PAK

**Keywords:** gland, hyperplasia, hypothyroidism, pituitary, diagnosis

## Abstract

Pituitary lesions can occur as a consequence of primary hypothyroidism and the biochemical imbalance associated with it, making its diagnosis a challenging task necessitating a thorough patient assessment by the treating physicians. We describe a young patient with pituitary hyperplasia due to primary hypothyroidism who presented with complaints of menstrual irregularities and weight gain. The patient was treated with thyroxine (T4) for primary hypothyroidism. The patient reported improvement in her symptoms along with the normalization of thyroid profile and interval reduction in the size of pituitary lesion on follow-up MRI scan.

## Introduction

Pituitary hyperplasia may or may not occur in patients with primary hypothyroidism as it has quite a variable incidence ranging from 25% to 81%. However, some evidence suggests that this might correlate to the high serum thyroid-stimulating hormone (TSH) level. Hence, one needs to remember this consequence of primary hypothyroidism while making the diagnosis [1,2]. The significance of making the right diagnosis of a pituitary mass cannot be emphasized more as it can save a patient from undue surgery or other interventions. In the presence of all current technological innovations in imaging, differentiating between different pituitary lesions remains a dilemma [3]. "Nipple sign" is the term used to describe the presentation of pituitary hyperplasia that looks like a midline homogenously enhancing mass with smooth edges, although this has also been noticed with other pituitary masses and is not exclusive to hyperplasia [3].

## Case presentation

A 23-year-old female presented with complaints of irregular menstrual cycles, primary infertility, and inability to lose weight over the last two years. Physical examination suggested no hirsutism, acne, infraclavicular fat pad, anterior neck swelling, or acromegalic features. Vital signs were all within normal limits with only a finding of a high BMI of 31 kg/m^2^.

**Figure 1:**
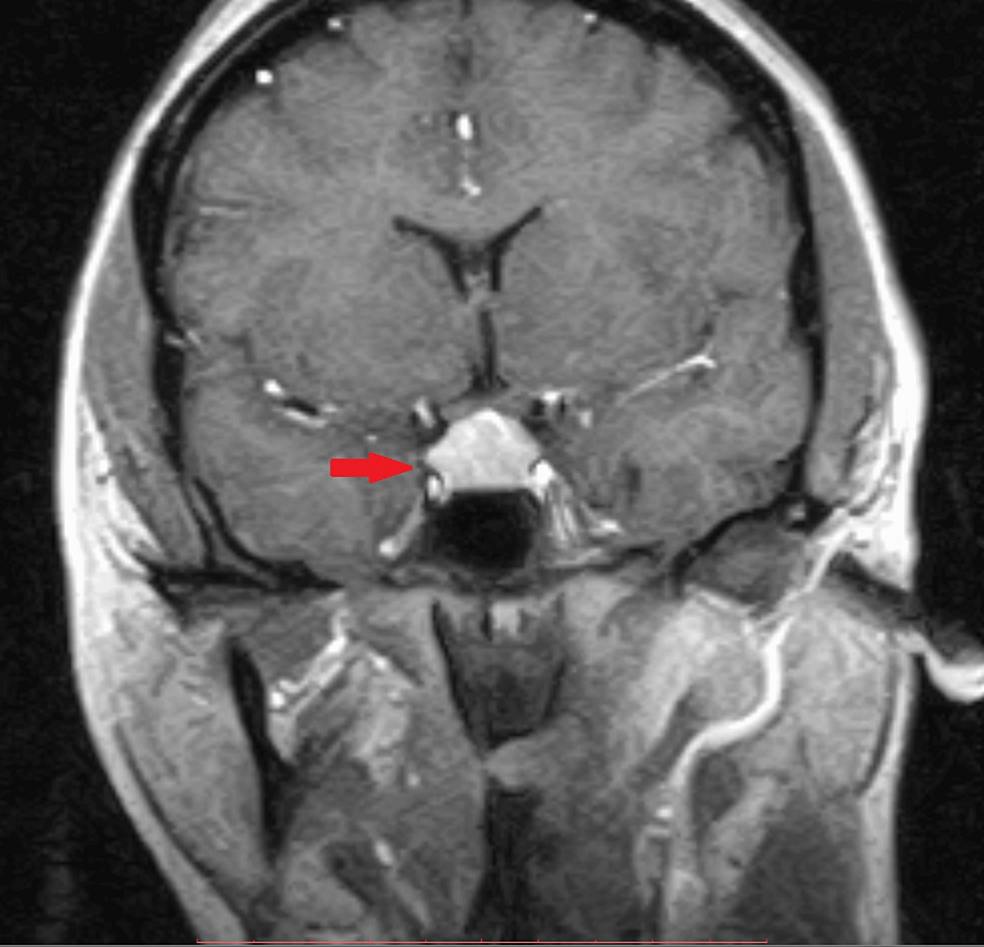
A coronal section at the level of the pituitary fossa, which demonstrates an enlarged, homogenously enhancing pituitary, causing the expansion of the pituitary fossa and extending into the suprasellar region, compressing upon the optic chiasm

The endocrine test panel revealed a high TSH, a low free thyroxine (T4), and a high prolactin level. Hence, pituitary imaging was done, which showed an enlarged pituitary mass of about 1.5 cm in size as shown in Figure 1.

Our patient did not mention any visual problems, and the field of vision was intact. Laboratory investigations done for our patient are shown in Table 1.

**Table 1:** Laboratory Investigations T4: thyroxine

Investigations	At presentation	Eight weeks later	Reference values
Random cortisol, mg/dL	12	-	8-16
Free thyroid hormone (T4), ng/dL	0.2	1.3	0.8-2.4
Thyroid-stimulating hormone, mIU/L	>150	4.2	0.27-4.20
Luteinizing hormone, IU/L	2	-	1.7-8.6
Follicle-stimulating hormone, IU/L	5.7	-	1.5-12.4
Prolactin, mIU/L	80	17	4-25
Insulin-like growth factor I, ng/mL	177	-	85-350

A patient had been visiting multiple doctors with her symptoms and had been advised of surgical resection of pituitary mass based on her MRI findings. This case was discussed in a multidisciplinary meeting with a neuroradiologist where it was decided that it was a homogenously enhancing pituitary likely representing a pituitary hyperplasia. The patient was started on treatment with thyroxine 100 mcg/day and was called for follow-up in 6-8 weeks with a repeat thyroid profile.

**Figure 2:**
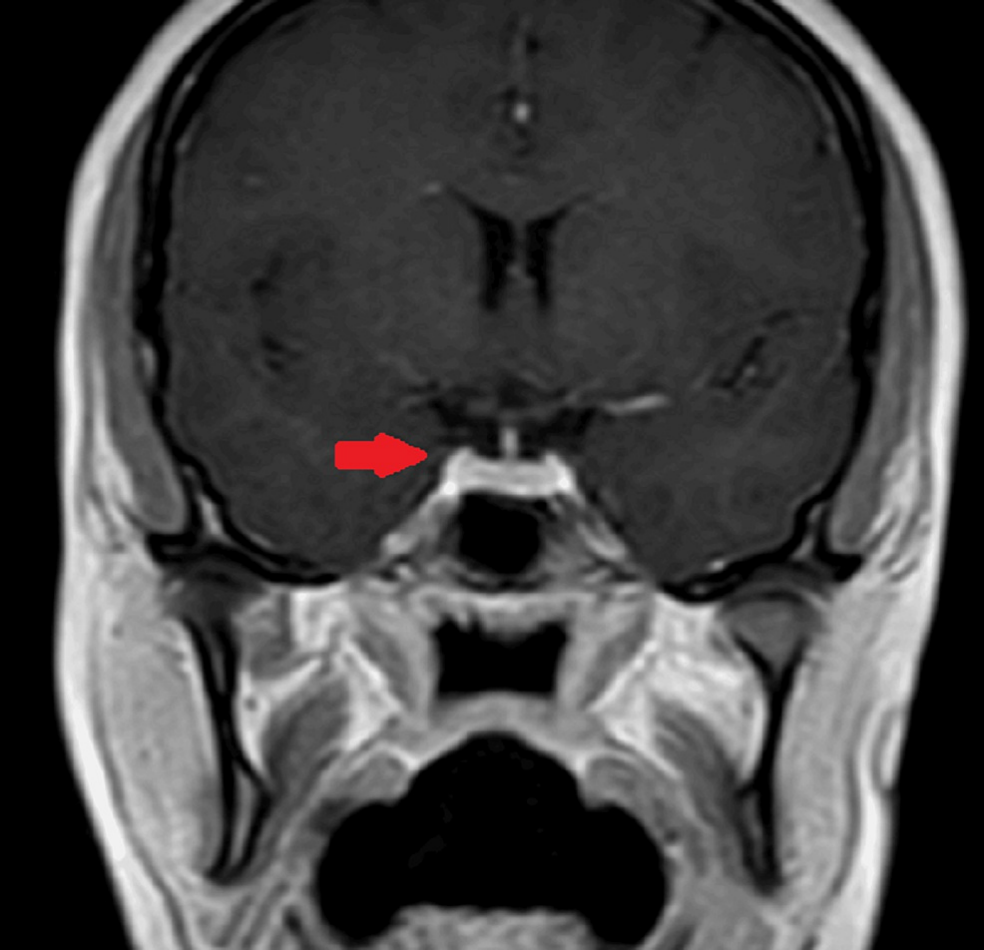
A coronal section showing treatment response with a significant reduction in the size of the pituitary mass

The patient reported improvement in her symptoms. Investigations showed the normalization of serum levels of TSH and free T4 levels. MRI done at three months was suggestive of interval reduction in the size of the pituitary gland with normal contours and no abnormal signal intensity seen in the pituitary fossa as shown in Figure 2.

## Discussion

Thyroxine deficiency causes a rise in trophic hormones via a negative feedback mechanism with a resultant pituitary hyperplasia. Pituitary hyperplasia secondary to primary hypothyroidism is the most frequent among these pituitary lumps, which accounts for about 33.3% of the total cases [3]. Primary hypothyroidism causes an increase in TSH-producing cells via a feedback loop bringing about change in pituitary anatomical structure [4]. Hyperprolactinemia may be noted in patients with primary hypothyroidism when there is a rise in thyrotropin-releasing hormone (TRH) via a negative feedback mechanism that augments prolactin secretion [5]. In young adults with this condition, presentation is usually with symptoms of hypothyroidism rather than those related to mass effect. Symptoms may range from stunted growth due to primary hypothyroidism itself to reduced growth hormone (GH) production [3].

Pituitary hyperplasia with primary hypothyroidism is well documented in the literature. However, our patient had been offered multiple treatment options including surgery before the right diagnosis was made. On average, response to optimal therapy for primary hypothyroidism is achieved in 85% of patients [6]. If a long-standing primary hypothyroidism is left untreated, it can lead to permanent damage to the pituitary gland, and a deficiency of one or more pituitary hormones can occur in patients [2]. Consideration for surgical intervention such as optic chiasm decompression should be reserved in patients developing visual symptoms or in obtaining a tissue diagnosis if the patient remains unresponsive to therapy [7].

The MRI scan we did for our patient showed a uniform enhancement of the pituitary gland with smooth contours on postcontrast images. The most common radiologic finding in primary hypothyroidism is a pituitary mass with suprasellar extension [7]. To differentiate between pituitary masses, the enhancing pattern on postcontrast scans plays a fundamental role. On unenhanced MRI scans, T1 images show areas of lower signal intensity for adenomas as compared to a normal gland, whereas a hyperplastic gland tends to enhance homogenously [8]. Pituitary macroadenomas especially larger lesions are often heterogeneous as different signal intensity is noted for different areas of cystic change or hemorrhage [8].

One important differential diagnosis of pituitary hyperplasia is TSH-secreting pituitary adenoma as both of these conditions have high levels of serum TSH; however, one is treated with tablets and the other with surgical resection [3]. Lymphocytic hypophysitis can also present as a pituitary mass on MRI. The other features noted with this include the likely absence of the normal posterior pituitary bright spot, dural enhancement, and a thickened infundibulum [9].

Patients treated with thyroxine having pituitary hyperplasia and hypothyroidism need interval monitoring with an MRI scan to ascertain the change in the pituitary lesion. If there is no reduction in size with thyroxine treatment, the diagnosis needs to be reconsidered [10]. Our patient had improvement in her symptoms within a couple of weeks after she was started on thyroxine therapy. Her MRI scan was done three months after thyroxine therapy with a significant reduction in the size of the mass. She had a complete normalization of her thyroid profile, and she conceived spontaneously in the following year. There were a few residual changes seen on MRI, which are likely the effects of untreated hypothyroidism that may or may not be seen indefinitely.

## Conclusions

The emphasis of our case lies on thorough biochemical investigations before interpreting imaging findings for endocrine diagnosis as pituitary masses may look the same but have varying endocrine implications. Pituitary hyperplasia is not a common diagnosis in our daily practice, but knowing about its presentation by an endocrinologist and a neuroradiologist can save a patient from unnecessary surgical intervention and cost associated with it.
